# Supplementary figures and images for: cAMP promotes acute lysosome biogenesis through TFEB nuclear import-export dynamics
Source: bioRxiv. 2025 Nov 26:2025.11.24.690233. Preprint. [Version 1] doi: 10.1101/2025.11.24.690233 (PMC12697359; doi:10.1101/2025.11.24.690233)

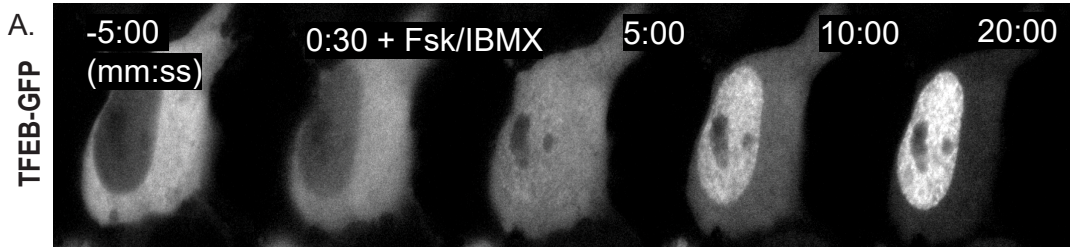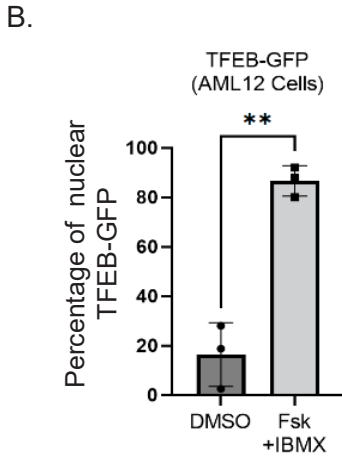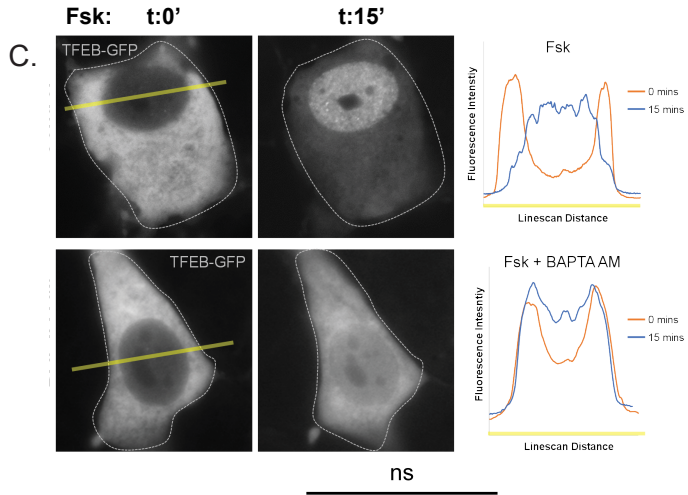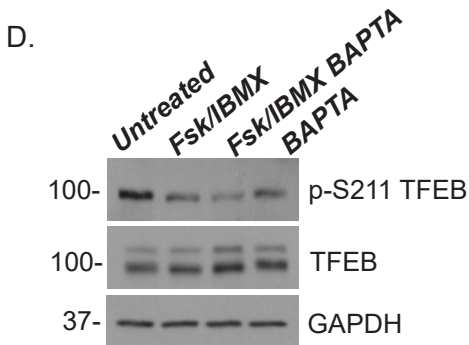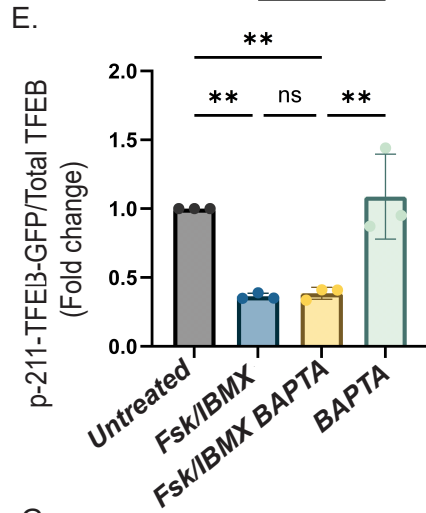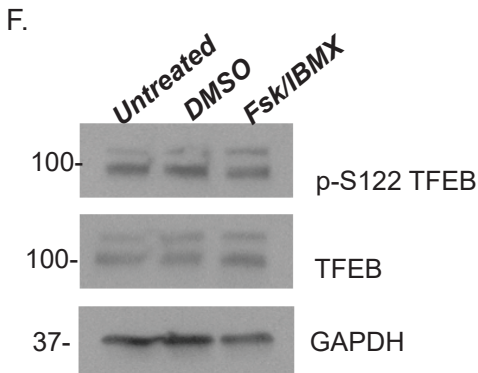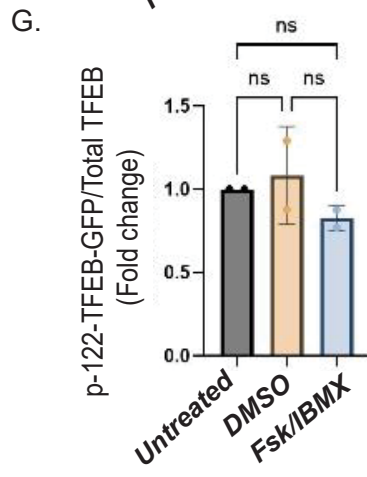

Supplement: Supplement 1 — Supplementary Figure 1. (A) Fluorescence micrographs of AML-12 cells transfected with TFEB-GFP show rapid nuclear translocation of TFEB following treatment with cAMP agonists (Fsk/IBMX). (B) Quantification of the nuclear-to-cytoplasmic TFEB-GFP ratio reveals a significant increase in nuclear localization within 30 minutes of treatment compared to DMSO control (n = 3; p < 0.05). This data suggests cAMP stimulates TFEB nuclear import in AML-12 cells. (C) Fluorescence micrographs of AML-12 cells transfected with TFEB-GFP and treated with cAMP agonists (Fsk/IBMX), with or without the calcium chelator BAPTA-AM. Chelation of intracellular calcium markedly reduces TFEB nuclear localization within 15 minutes. (D) Quantification of the nuclear intensity of TFEB-GFP confirms a decrease in nuclear TFEB levels in the BAPTA-AM-treated group compared to control. This data suggests cAMP-stimulated calcium promotes TFEB nuclear import in AML-12 cells. (E) Western blot analysis of whole-cell lysates from HeLa cells treated with DMSO (30 min), Fsk/IBMX (30 min), or with or without BAPTA-AM. Blots were probed with a phospho-specific antibody against TFEB S211. (F) Densitometric analysis of panel (E) phospho-S211(normalized to GAPDH) showed reduced S211 phosphorylation in Fsk/IBMX condition and BAPTA-AM but no change following Fsk/IBMX treatment compared to BAPTA-AM condition (n = 3, one-way ANOVA). (G) Western blot analysis of whole-cell lysates from HeLa cells treated with DMSO (30 min), Fsk/IBMX (30 min), or left untreated. Blots were probed with a phospho-specific antibody against TFEB S122. (H) Densitometric analysis of panel (G) phospho-S122 (normalized to GAPDH) showed no change following Fsk/IBMX treatment compared to DMSO control (n = 3, one-way ANOVA). [file media-1.pdf]

A.

Fsk/IBMX

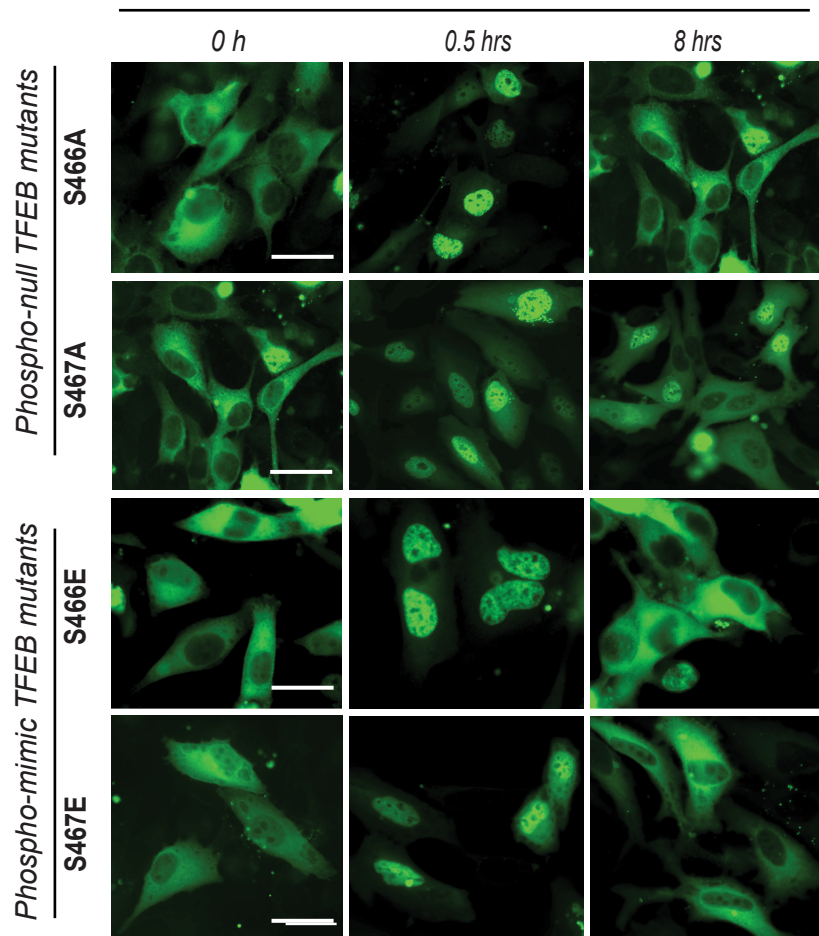

B.

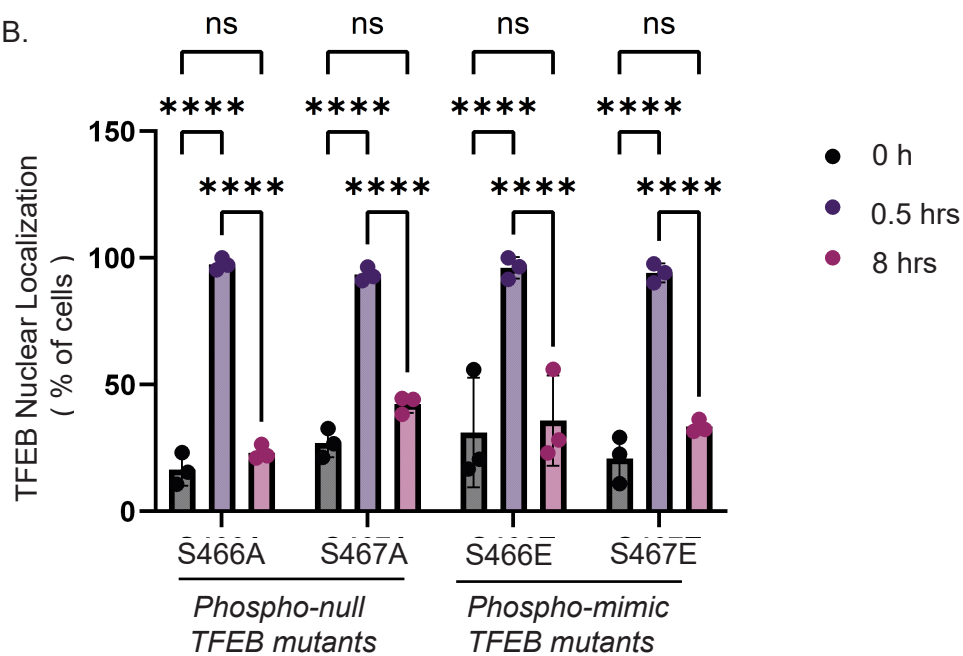

Supplement: Supplement 2 — Supplementary Figure 2. (A) Fluorescence micrographs of HeLa cells transiently expressing TFEB–GFP phospho-null mutants (S466A, S467A) or phospho-mimetic mutants (S466E, S467E) treated with Fsk (10 μM) and IBMX (0.5 mM) for 30 minutes or 8 hours. (B) Quantification of nuclear-to-cytoplasmic TFEB–GFP ratios corresponding to panel A. All mutants display robust nuclear retention at 30 minutes compared with baseline (0 hour). By 8 hours, phospho-null (S466A, S467A) and phospho-mimetic (S466E, S467E) mutants show predominant cytoplasmic localization similar to wild-type TFEB, although a modest degree of nuclear retention persists in the phospho-null forms. Data represent mean ± SEM from three independent experiments (n = 3; 100 cells per condition; one-way ANOVA with Tukey’s multiple-comparison test;****P < 0.0001). [file media-2.pdf]

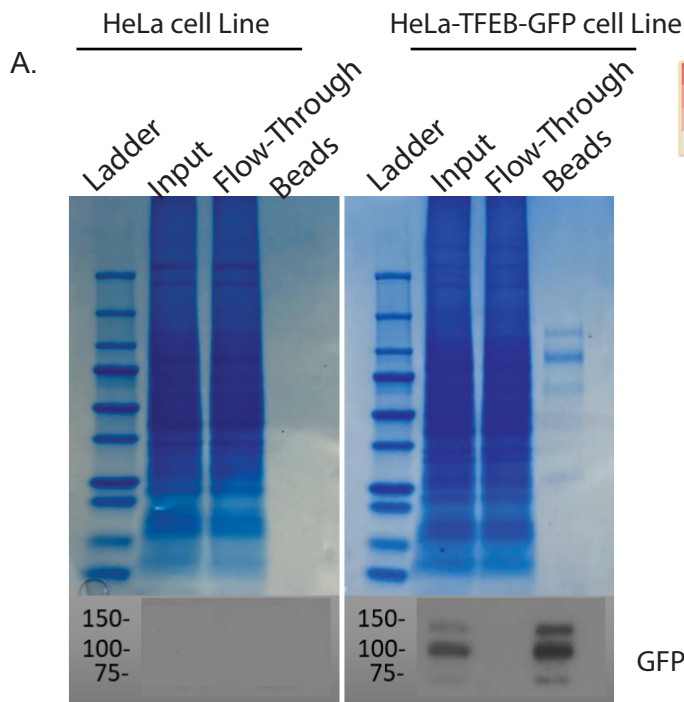

**B. Import Phase**

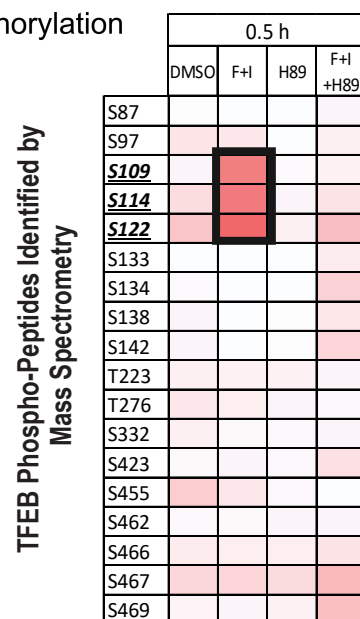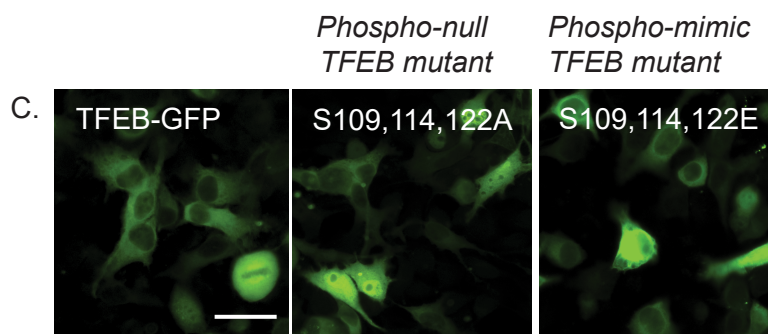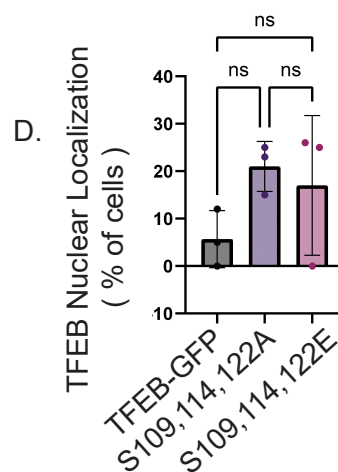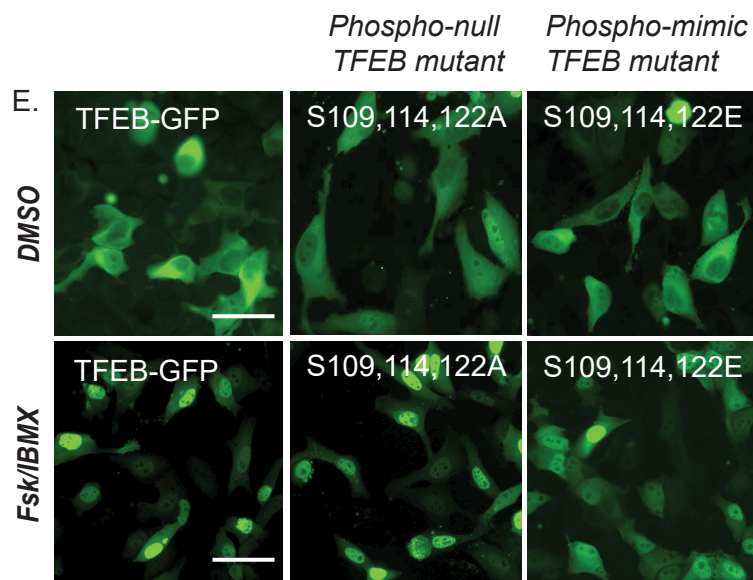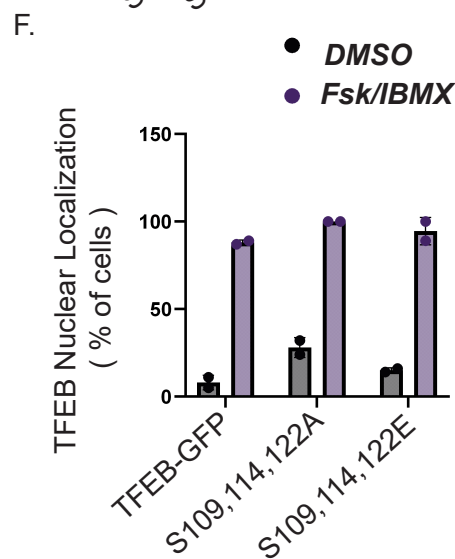

Supplement: Supplement 3 — Supplementary Figure 3. (A) Coomassie blue stain and GFP western blot showed a successful demonstration of GFP-Trap isolation from HeLa cells stably expressing TFEB-GFP versus normal HeLa, where no pulldown artifacts were observed. (B) Heatmap of TFEB–GFP phosphopeptides identified by GFP-Trap pulldown from HeLa cells treated for 30 minutes with DMSO, Fsk (10 μM) + IBMX (0.5 mM), Fsk/IBMX + H89, or H89 alone (50 μM), analyzed by mass spectrometry (n = 3 independent experiments). Phosphorylation at S109, S114, and S122 was significantly increased during the nuclear import phase in a PKA-dependent manner. (C) Fluorescence microscopy of HeLa cells expressing basal TFEB–GFP phospho-null (S109,114,122A) or phospho-mimetic (S109,114,122A) mutants. (D) Quantification of nuclear TFEB levels from panel B shows no significant differences in basal nuclear localization between mutants (n = 3; 100 cells per condition; one-way ANOVA). (E) Representative micrographs of HeLa cells expressing TFEB mutants and treated with DMSO or Fsk + IBMX for 30 min. cAMP stimulation increased nuclear TFEB in both phospho-null and phospho-mimetic mutants. (F) Quantification of nuclear TFEB confirms a significant increase upon Fsk/IBMX treatment compared with DMSO (n = 2; 100 cells per condition). These data indicate that S109, S114, and S122 are not essential for cAMP-mediated TFEB nuclear import. [file media-3.pdf]
